# DHA and Its Elaborated Modulation of Antioxidant Defenses of the Brain: Implications in Aging and AD Neurodegeneration

**DOI:** 10.3390/antiox10060907

**Published:** 2021-06-03

**Authors:** Mario Díaz, Fátima Mesa-Herrera, Raquel Marín

**Affiliations:** 1 Laboratory of Membrane Physiology and Biophysics, Department of Animal Biology, School of Biology, Universidad de La Laguna, 38206 Tenerife, Spain; fmesaher@ull.edu.es; 2 Instituto Universitario de Enfermedades Tropicales y Salud Pública de Canarias (IUETSP), Universidad de La Laguna, 38206 Tenerife, Spain; 3 Unidad Asociada ULL-CSIC “Fisiología y Biofísica de la Membrana Celular en Enfermedades Neurodegenerativas y Tumorales”, 38206 Tenerife, Spain; rmarin@ull.edu.es; 4 Laboratory of Cellular Neurobiology, Department of Basic Medical Sciences, School of Medicine, Universidad de La Laguna, 38206 Tenerife, Spain

**Keywords:** docosahexaenoic acid (DHA), lipid–phospholipid peroxidation, indirect antioxidants, glutathione peroxidase 4, gene transcription, intron retention, neuroprotection, Nrf2, selenium, aging, Alzheimer’s disease

## Abstract

DHA (docosahexaenoic acid) is perhaps the most pleiotropic molecule in nerve cell biology. This long-chain highly unsaturated fatty acid has evolved to accomplish essential functions ranging from structural components allowing fast events in nerve cell membrane physiology to regulation of neurogenesis and synaptic function. Strikingly, the plethora of DHA effects has to take place within the hostile pro-oxidant environment of the brain parenchyma, which might suggest a molecular suicide. In order to circumvent this paradox, different molecular strategies have evolved during the evolution of brain cells to preserve DHA and to minimize the deleterious effects of its oxidation. In this context, DHA has emerged as a member of the “indirect antioxidants” family, the redox effects of which are not due to direct redox interactions with reactive species, but to modulation of gene expression within thioredoxin and glutathione antioxidant systems and related pathways. Weakening or deregulation of these self-protecting defenses orchestrated by DHA is associated with normal aging but also, more worryingly, with the development of neurodegenerative diseases. In the present review, we elaborate on the essential functions of DHA in the brain, including its role as indirect antioxidant, the selenium connection for proper antioxidant function and their changes during normal aging and in Alzheimer’s disease.

## 1. Introduction

The brain is one of the richest organs in lipids, just after adipose tissue. In nerve cells, lipids represent approximately 60–70% of total brain constituents. The brain lipidome is extremely complex, with more than 15,000 different molecular species, mostly concentrated in cellular membranes [1,2]. However, despite this great diversity, approximately 50% of brain membranes (though variation between brain areas and cell types) are composed of a single group of lipids, namely polyunsaturated fatty acids (PUFAs). Chemically, PUFAs are lipid species defined by their number of double bonds (>3) and the position of the first unsaturation with respect to the methyl end of the aliphatic chain, giving rise to two major families of PUFAs, i.e., the n-3 and n-6 series. Most PUFAs in the brain contain 18 or more carbon atoms and are accordingly named long-chain PUFAs or LCPUFAs, of which the most abundant are arachidonic acid (AA, 20:4n-6) and docosahexaenoic acid (DHA, 22:6n-3). Because of their elevated number of unsaturations, they are also referred to a highly unsaturated fatty acids (HUFAs), and this property has an enormous impact on their cellular biology. DHA levels in nerve cell membranes are generally much higher (15–50% of total fatty acids, depending on the cell type lineage) than those of AA (2–5%) [3,4,5,6,7]. Within nerve cell membranes, DHA esterifies the *sn*-2 position of glycerophospholipids, particularly phosphatidylethanolamine (PE), the most abundant phospholipid in nerve cells. 

The abundance of PE-containing DHA in nerve cells indicates that this molecular association is a significant determinant of the structural and physicochemical properties of cell membranes. Intrinsic membrane properties such as microviscosity, passive permeability, phase separation and microdomain segregation, lateral mobility, lipid–protein and protein–protein interactions and conformational transitions of membrane proteins have all been shown to be modulated by DHA [3,5,6,8,9]. This influence on membrane properties is due to the fact that DHA impacts the bilayer’s physicochemical state as it has conformational properties that remain highly structured but fluid membranes capable of accommodating rapid protein conformational changes [5,10]. Moreover, the mutual aversion of DHA and cholesterol drives the lateral segregation of DHA-containing phospholipids into highly disordered domains away from cholesterol-enriched ordered domains, which largely support the uneven distribution of membrane functions (synaptic densities, signalosomes, protein clusters, signaling events, neurotransmission and electrical conduction, amongst others) [5,6,10,11,12,13,14,15]. Particular attention has been given to cholesterol-enriched ordered domains, which are ordinarily enriched in saturated sphingolipids and specific scaffold proteins, named lipid rafts, which serve as signaling platforms [6,10,16]. Both domains are compositionally and organizationally opposite and contain different subsets of integral proteins, which give them different physiological roles in nerve cells [11,12,14,17].

Research on the actions of DHA in the brain has been growing steadily over the last three decades, and it is now widely held that its biological effects extend well beyond the initial conception of essential structural properties. In the next sections we review and discuss the diversity of physiological effects demonstrated for DHA, its novel role as an indirect antioxidant, its changes with aging, and the relation between its dyshomeostasis and neurodegenerative diseases, specifically in Alzheimer’s disease.

## 2. DHA Is a Pleiotropic Molecule in Nerve Cells

Further to its structural role, DHA is involved in neurodevelopmental processes, including neurogenesis, neuritogenesis, synaptogenesis, neuronal differentiation and axonal growth and regeneration [4,18,19,20,21,22,23]. DHA is essential for brain homeostasis not only during development, but also as a neuromodulator of nerve cell function throughout the lifespan. Indeed, formation and differentiation of novel neurites and synapses, refinement of synaptic connectivity, neurotransmitter release and memory consolidation processes are, at different levels, modulated by DHA [3,4,18,19,21,23,24,25].

The relevance of DHA to brain health is supported by a number of epidemiological and experimental studies associating its depletion with the development of neurodegenerative diseases [7,26,27,28,29,30,31] as well as in psychiatric disorders such as major depression or bipolar disorder [32,33,34]. Furthermore, a number of experimental studies have demonstrated neuroprotective effects for DHA in different models of acute brain injury (ischemic stroke, focal cerebral ischemia, ischemia/reperfusion and intracerebral hemorrhage) [35,36]. These studies have shown that DHA protects neurons and astrocytes within the infarcted areas [35,37]. Interestingly, DHA rescues neurons indirectly by protecting astrocytes and by downregulation of microglial activation in the infarcted portions [35]. The astrocyte-triggered neuroprotection in response to ischemic brain injury appears to occur through secretion of growth and neurotrophic factors, such as brain-derived neurotrophic factor (BDNF) [38] or lipid mediators; docosanoids, such as neuroprotectin D1 (NPD1) [4,39,40] and synaptamide (N-Docosahexaenoylethanolamine) [23], or is followed by activation of survival signaling pathways against oxidative and proinflammatory insults, such as activation of the PI3K/Akt pathway or inhibition of NF-κB activation and COX-2 expression [39,41,42]. In parallel, DHA has been recently documented to prevent uncontrolled oxidative stress (UOS) by upregulating expression and activity of cellular antioxidant proteins belonging to the glutathione and thioredoxin antioxidant systems [43,44,45,46], which, as discussed below, is the gold standard strategy for brain lipid homeostasis. 

Further, DHA and NPD1 have been shown to modulate apoptotic induction in different models of neurodegeneration (reviewed in [4,40,47,48,49,50,51]). For instance, exposure to oxidative challenge, intraventricular infusion of Aβ peptides, induction of excitotoxicity or ischemia/reperfusion maneuvers all appear to share a common pattern of enhancement of pro-apoptotic proteins Bik and Bax, which is totally or partially counteracted by treatments with DHA or NPD1 [4,41,49,50,51,52]. On the other hand, anti-apoptotic proteins Bcl-2, Bcl-xl and Bfl-1 were enhanced by DHA and docosahexaenoic-acid-derived mediators, including the recently discovered very-long-chain DHA-derived mediators elovanoids, such as ELV-N32 and ELV-N34 [47]. Notably, in general, NPD1 promotes a much larger increase in the anti-apoptotic Bcl-2 family of proteins than DHA itself [39,40,41,47,52]. Overall, the current view is that induction of anti-apoptotic Bcl-2 family members by DHA and related bioactive metabolites breaks the mechanistic link between proinflammatory signaling, cell-damaging events and apoptosis triggered by multiple noxious stimuli, which largely determines the survival of aged and terminally differentiated nerve cells. A summary of functions demonstrated for DHA in brain biology is shown in Table 1.

## 3. The Hostile Environment of Brain Parenchyma for LCPUFAs

**Figure 1:**
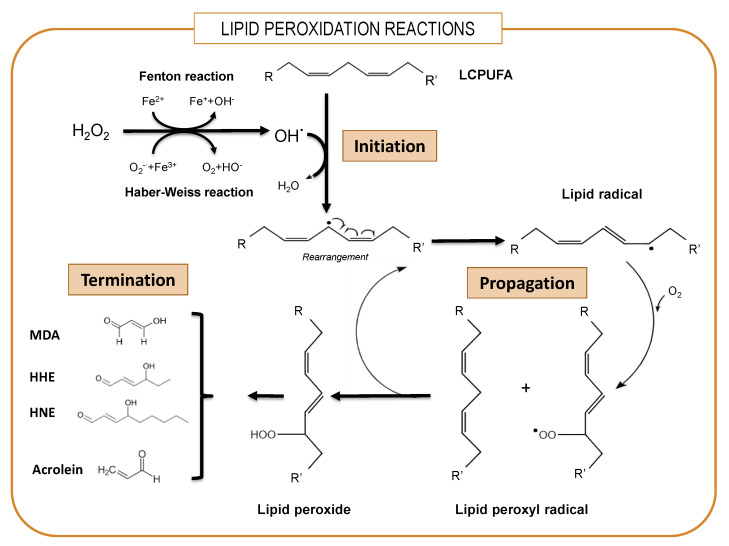
Simplified overview of lipid peroxidation. The lipid peroxidative process involves sequential phases of initiation, propagation and termination. LCPUFAs: long-chain polyunsaturated fatty acids. MDA: malondialdehyde. HNE: 4-hydroxy-2-trans-nonenal. HHE: 4-hydroxy-2-trans-hexenal. Acrolein: 2-propenal.

One paramount feature of brain physiology is its very high metabolic rate and elevated oxygen consumption, which inevitably produces amounts of reactive oxygen and nitrogen species (ROS and RNS, respectively) as by-products [65]. This includes the highly reactive superoxide anion O_2_^−^ that is converted to H_2_O_2_ by the ubiquitous enzyme superoxide dismutase. In addition, the brain is especially rich in redox transition metals, particularly iron and copper. In the presence of iron, Fe(II) reacts with endogenous H_2_O_2_ to produce Fe(III) as well as the highly reactive hydroxyl radical OH^•^ or peroxynitrite (ONOO^-^) by virtue of the Fenton reaction at the expense of endogenous reducing agents. These highly oxidant radicals easily react with polyunsaturated lipids, forming lipid radicals (L^•^), which in the presence of O_2_ form lipid peroxyl radicals (LOO^•^) and lipid peroxide (LOOH). This phase is self-propagating and self-sustaining because as LOO^•^ reacts with other PUFAs, it generates additional L^•^, which, in turn, forms surplus LOO^•^ and LOOH in a true chain reaction [66] (Figure 1). Lipid peroxidation is a particularly detrimental cycle, as reaction products can also act as triggers for the generation of additional lipid peroxides in the membrane via lipoxygenases, which catalyze the oxygenation of polyunsaturated fatty acyl groups to hydroperoxides [67]. Finally, as a result of electron rearrangements in the degradation phase, products of lipid peroxidation undergo fragmentation and yield different reactive intermediates called reactive carbonyl species (RCS) such as malondialdehyde (MDA), unsaturated aldehydes such as 4-hydroxy-2-*trans*-nonenal (HNE) and 4-hydroxy-2-*trans*-hexenal (HHE) and 2-propenal (acrolein) [68]. These RCS react with histidine, lysine and cysteine residues in proteins through Michael addition, converting stable adducts with carbonyl functionalities into proteins and altering structural, catalytic and transport proteins [65]. Obviously, as most polyunsaturated lipids in the brain are integrated into phospholipids, their peroxidation causes structural damage of membranes, thereby severely affecting domain viscosity, passive permeability and lipid–protein and protein–protein interactions, which impact neurotransmission, signal transduction, ion transport and electrical conduction [66,67,68,69,70,71].

Another important aspect that makes the brain especially susceptible to oxidative damage is that its contents of antioxidant enzymes and related activities are relatively low compared to other tissues [72]. For instance, in mouse brain the specific activity of cytosolic glutathione peroxidase (GPx1) is less than 5% that of the kidney and liver, and that of glutathione reductase represents only 32% and 65% that of the kidney and liver, respectively [73]. On the other hand, concentration of the tripeptide glutathione, γ-L-Glutamyl-L-cysteinylglycine, used as the main redox metabolite, is remarkably low in nerve cells and becomes even lower with aging [72]. 

Overall, the concurrency of all these factors renders brain parenchyma extremely vulnerable to free radical-induced peroxidation of LCPUFAs and particularly of DHA [45,72].

## 4. Fighting Against Lipid Peroxidation in the Brain

**Figure 2:**
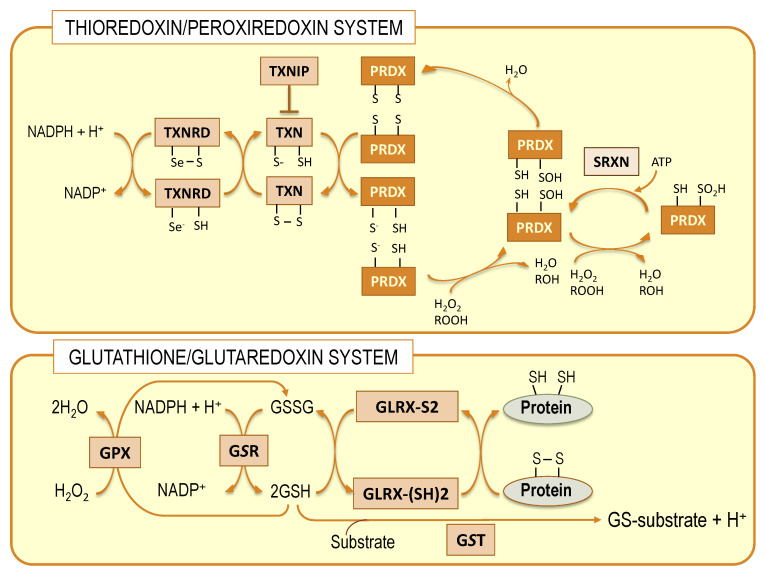
Schematic representation of thioredoxin/peroxiredoxin and glutathione/glutaredoxin systems. Both systems are interconnected at different levels. TXN: thioredoxin; TXNRD: thioredoxin reductase; TXNIP: thioredoxin interacting protein; PRDX: peroxiredoxin; SRXN: sulfiredoxin; GPX: glutathione peroxidase; GSR: glutathione *S* transferase; GLRX: glutaredoxin.

Nerve cells are equipped with different antioxidant systems that provide a degree of protection against oxidative damage caused by lipid peroxides [74,75]. Given the aforementioned scenario, a paramount question arises on how DHA can be present in high amounts in brain phospholipids without being oxidized in such adverse conditions. Recent experimental evidence is just starting to decipher this enigma. It is known that nerve cells are endowed with different antioxidant systems that render them resistant to oxidative damage. This antioxidant capacity is accomplished by phase II detoxifying enzymes from thioredoxin/peroxiredoxin and glutathione/glutaredoxin systems (Figure 2). 

These systems coexist in nerve cells with the classical superoxide dismutase/catalase system and make use of hydrophilic thiol-containing molecules, thioredoxin and glutathione, respectively, as electron donors to produce conjugated metabolites [76]. Members of the thioredoxin system, specifically thioredoxin reductases, are capable of reducing some non-disulfide-containing molecules, such as lipid hydroperoxides, independently of thioredoxin [74,77]. However, it is only in the glutathione system that isoenzymes exist, specifically the glutathione peroxidase 4 (GPx4) family, members of which are capable of recovering oxidized membrane lipids in situ [45,78,79]. The relevance of these enzymes in nerve cells is significant since they provide a direct mechanism to preserve neuronal membrane integrity [44,45]. 

## 5. The Central Role of GPx4 in the Protection of Membrane LCPUFAs from Peroxidation

**Figure 3:**
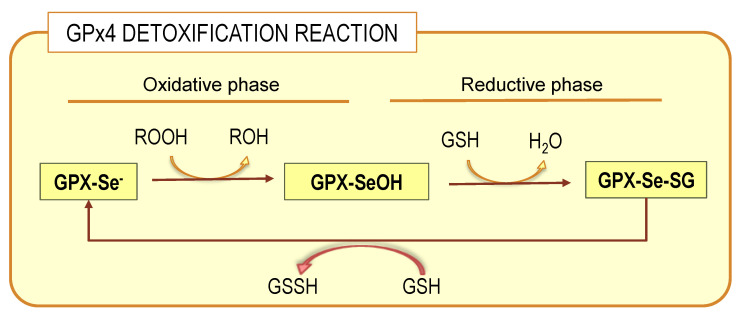
GPx4 detoxification reaction. The complete process involves two phases: oxidative and reductive connected by the selenenic acid intermediate (GPX-SeOH), which is reduced at the expenses of glutathione (GSH). For details on whole process, see the main text.

Glutathione peroxidases (GPxs) are a family of selenoproteins (selenoP) ubiquitously expressed along animal phylogeny. Eight different GPx isoenzymes have been described GPx1-8, each with unique characteristics that determine their precise biological role [80]. All GPxs bear selenocysteine, an active amino acid, as their catalytic domain, which provides them with a more efficient reaction with peroxide substrates [80]. Phospholipid hydroperoxide glutathione peroxidase, glutathione peroxidase 4 or GPx4, is the most widely expressed isoform in brain tissue, existing as a momomeric membrane-anchored glycoprotein [80]. It is unique between glutathione peroxidases in that is capable of reducing complex lipid peroxides, such as phospholipid hydroperoxides, even when integrated in highly structured lipid–protein assemblies such as lipoproteins and membranes, without prior action of membrane phospholipase A2 [79,81,82]. GPx4 preferentially uses glutathione as an electron donor as long as cellular concentrations of the reduced form (GSH) but may accept other thiol groups in proteins as a reduced equivalent. Therefore, GPx4 can either act as a GSH peroxidase or a thiol peroxidase depending on the availability of GSH [83]. Mammalian cells, including nerve cells, include three different GPx4 isoenzymes, namely cytoplasmic, nuclear and mitochondrial isoforms, which differ in their N-terminal sequences [12,31]. The detoxification reaction carried out by GPx4 encompasses two independent events: a first oxidation of the reduced enzyme by hydroperoxide (ROOH) and a second reduction of the oxidized enzyme by GSH (Figure 3). 

In the oxidative phase of the catalytic cycle, the selenolate at the active site is oxidized by the hydroperoxide to form selenenic acid derivative (GPx-SeOH). The second part of the cycle is more complex. The overall reduction of the oxidized selenium proceeds by two subsequent steps; the first requires a GSH molecule and yields the glutathionylated enzyme, that is, a selenodisulfide. In the second reduction step, a second GSH molecule forms the stable end product oxidized glutathione, GSSG, and releases the reduced selenium for the next cycle. It has been observed that the capacity of GPx4 to detoxify hydroperoxides occurs both for free-state hydroperoxides or those bound to membrane phospholipids [81]. This ability of GPx4 is due to the presence of a large hydrophobic surface that allows close association with structured lipid–protein assemblies from lipoproteins to membranes [82,84]. 

## 6. The Elaborated Transcriptional Regulation of the *Gpx4* Gene by DHA

**Figure 4:**
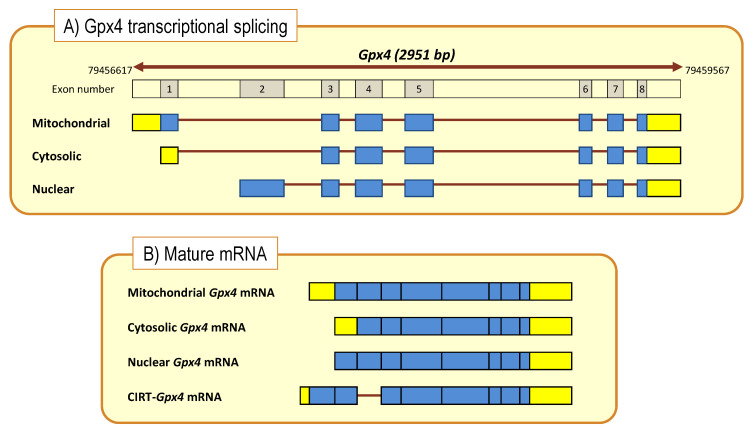
*Gpx4* gene structure and transcriptional processing (**A**) to generate mature and CIRT mRNAs (**B**). Boxes indicate coding exons (blue) and UTRs (yellow). Bold brown lines represent intronic sequences. Cytoplasmic intron-sequence-retaining transcripts (CIRTs) derived from Gpx4 gene.

From the molecular point of view, mammalian GPx4 comprises three isoenzymes found in the cytoplasm (c-GPx4), nucleus (n-GPx4), mitochondria (m-GPx4) and also in the endoplasmic reticulum of mammalian cells. All these isoforms are very similar but differ in their N-terminal sequences. GPx4 isoenzymes derive from a single gene, *Gpx4*, by alternative splicing [79,80,81]. The *Gpx4* gene comprises eight exons, of which exons 3-8 encode for the functional enzyme are identical in the three isoforms. The differential N-terminal sequences are due to three starting sites for translation (Figure 4) in two alternative exons 1 (E1a and E1b). Exon 1a contains two in-frame translational starting sites (5′AUG and 3′AUG) separated by a sequence that encodes for a mitochondrial leader peptide. Translation initiation at the 5′AUG produces the m-GPx4 isoenzyme, while translation from 3′AUG (which lacks the leader signal) yields c-GPx4. Because the mitochondrial leader peptide is cleaved off after import into mitochondria, c-GPx4 and m-GPx4 contain identical primary structures. On the other hand, the alternative first exon (E1b) encodes the N-terminal part of the nuclear isoform, n-GPx4, and contains a nuclear targeting sequence, which is retained after nuclear import and hence differs from the protein sequence of m/c-GPx4 [81,84,85]. 

Studies in nerve cells have revealed that the three isoforms coexist in the same cell and that the absolute abundance of splice variants vary depending on the biological condition and the isoform considered. Generally, the abundance sequence as determined in cell extracts from different murine septal and hippocampal preparations is c-GPx4 (~3-fold) >> m-GPx4 > n-GPx4, but their relative amounts vary depending on the bioavailability of DHA [43,44]. 

Recent studies on these experimental models have revealed that DHA modulates *Gpx4* gene expression in hippocampal cells, affecting transcripts of cytosolic, mitochondrial and nuclear isoforms [43,44]. Results from hippocampal HT22 cells show that changes induced by DHA are specific to *Gpx4* since no variations were detected for other *Gpx* genes, such as the ubiquitous cytosolic *Gpx1* gene. Of note, DHA-induced upregulation affects gene splicing to different extents, the absolute change for the cytoplasmic transcript *c-GPx4* being about 5 times larger than for *m/n-GPx4* transcripts. In parallel to these changes, DHA treatment also increases GPx4 (and total GPX) activities shortly after DHA exposure, following a time course compatible with the delay needed for Gpx4 mRNA translation. Interestingly, these effects were specific to DHA and were not mimicked in arachidonic acid [43]. 

In vivo studies also show that nutritional interventions using diets with different supplies of DHA modify *Gpx4* transcriptional activity in the hippocampus. Thus, C57BL/6 mice fed experimental diets containing or lacking DHA display differential *Gpx4* transcriptional regulation. Hence, absolute quantification of *Gpx4* isoforms revealed that low-DHA diets leads to the stimulation of gene expression of all isoforms, with the cytosolic isoform showing the largest stimulation [44]. These observations are physiologically relevant and suggest a compensatory genetic strategy to ensure protection of essential DHA from oxidative damage under conditions of very limited DHA availability. By increasing *Gpx4* mRNA expression and GPx4 protein biosynthesis, cellular resistance to oxidative damage of DHA-containing phospholipids is ensured.

Another striking feature of DHA regulation of the *Gpx4* gene in hippocampal cells was the transcriptional enhancement of an unexpected transcript, the sequence of which retained the intron I1b located between exons E1b and E2 (Figure 4). This sequence, named *I-Gpx4*, was detected in HT22 cells as well as in the hippocampus of C57Bl/6 mice [44], and was indeed more abundant than *n-Gpx4* (3-fold on average) but less than *c/m-Gpx4*. Intron retention as genetic strategy has been gaining consistency in the last decade, as an alternative processing of conventional (nuclear) mRNA in the cytoplasm, adding a novel level of complexity in gene expression regulation. These sequences, known as cytoplasmic intron-sequence-retaining transcripts (CIRTs), comprise small fractions of introns within specific genes [86]. In recent years, a number of transcripts containing intronic sequences and subjected to cytoplasmic splicing have been discovered for a number of genes in different cell lineages [86]. In this sense, a recent study performed in mouse brains revealed that nearly 60% of total gene transcripts in dendrites correspond to CIRTs [87], which strengthens the widespread appearance of this alternative splicing. Examples of intron retention in transcripts of relevance in neuronal cells include the *KCNMA1* gene (encoding for BK channels [88,89], *GABRG3* gene (encoding for GABA-A receptor γ-subunit) [86], *GRIN1* gene (encoding for type 1 NMDA receptor) [86], *CACNA1H* gene (encoding for T type α-1 subunit voltage-dependent Calcium channel) [90] and *Calm3* gene (encoding for calmodulin-3) [91], and the list of genes is growing steadily. 

The neurobiological relevance of CIRTs as an alternative mechanism of post-transcriptional gene regulation is suggested by their abundance in dendrites. As CIRT processing in dendrites occurs by extranuclear splicing, the clear advantage emerges that mRNA processing and protein translation proceeds without the need for shuttling molecules to, and from, the neuronal soma [86,92]. Further, such dendritic mRNA localization and processing enables neurons to alter the synaptic proteome to induce plastic changes in response to synaptic stimuli involved in long-term synaptic plasticity, essential for learning and memory. In this sense, individual synapses become decision-making units, controlling gene expression in a nucleus-independent and spatially and temporarily restricted manner [93]. Dendritic targeting of mRNAs is achieved by microtubule-dependent transport of mRNAs, which are packaged into large ribonucleoprotein (RNP) particles containing an array of trans-acting RNA-binding proteins [93] including the minor spliceosome [94]. In context of the present article, a plausible scenario for this stimulus-dependent cytoplasmic splicing of *I-Gpx4* in hippocampal cells would locally and rapidly enhance the antioxidant capacity against the deleterious attack of lipid peroxides produced by local oxidative insults, which would ensure the integrity of synaptic phospholipids. 

Interestingly, the expression level of *I-Gpx4* is subjected to significant upregulation by DHA treatment. In absolute terms, DHA treatment leads to a 7-fold increase of *I-Gpx4* compared to unstimulated HT22 cells [44], pointing to a significant adaptive role of this isoform in hippocampal cells. In a different paradigm, C57Bl/6 mice receiving a low-DHA diet displayed higher expression levels of *I-Gpx4* in the hippocampus compared to animals receiving a high-DHA diet. These data suggest that critical DHA levels or its deficient supply is accompanied by an increase in *I-Gpx4*, as part of a compensatory strategy to preserve DHA-enriched phospholipids [44].

## 7. Transcriptional Regulation of Brain Antioxidant Defense by DHA: Beyond GPx4 

Transcriptional regulation of brain antioxidant defense by DHA also affects other members of the glutathione/glutaredoxin system as well as the thioredoxin/peroxiredoxin system (Figure 2) [43,44]. Thus, supplementation of cultures with DHA to hippocampal cells brings about the upregulation of *Gsr* (encoding for glutathione reductase) and *Gclc* (encoding for the catalytic subunit of glutamate-cysteine ligase). This was accompanied by increased cytosolic glutathione reductase activity and levels of total glutathione [43,44]. Furthermore, regarding the thioredoxin/peroxiredoxin system, DHA exposure to hippocampal HT22 cells upregulates the expression of the genes encoding for cytoplasmic thioredoxin and thioredoxin reductase (*Txn1* and *Txnrd1*) as well as their mitochondrial counterparts (*Txn2* and *Txnrd2*). These changes were followed by an equivalent increase in total thioredoxin reductase activity. Further, within this same system, DHA also upregulates the expression of 2-Cys type peroxiredoxin genes, namely *Prdx2* (encoding for one of the three cytosolic peroxiredoxins, PRDX2) and *Prdx3* (encoding for the only mitochondrial peroxiredoxin, PRDX3), as well as the *Srxn1* gene (encoding for sulfiredoxin, SRXN). Compatible results for DHA regulation of genes from the thioredoxin/peroxiredoxin system were obtained in vivo in mice hippocampus, though upregulated genes varied depending on the genotype (i.e., wild type or transgenic APP/PS1) and the differential DHA supply [43]. The relevance of these observations to the brain is enormous since mammalian thioredoxin reductases (TrxRs) can reduce some non-disulfide-containing molecules, including lipid hydroperoxides and other organic hydroperoxides, even when levels of reduced thioredoxin are limited [95]. Moreover, peroxiredoxins act as hydroperoxide scavengers by oxidation of one Cys residue in the PRX’ catalytic center to produce sulfenic acid (Cys-SOH). As typical PRDXs are 2-Cys, the first Cys-SOH then reacts with the second thiol group to form a disulfide bridge [95,96,97]. Regeneration of the reaction center of PRDX occurs upon oxidation of thioredoxin (Figure 2). Further, under sustained oxidative conditions PRDXs cannot be fully regenerated but are further oxidized to sulfinic acid (Cys-SO_2_H), which inactivates the enzyme. However, oxidized 2-Cys PRDXs may still be reactivated by sulfiredoxin upon hydrolysis of ATP [98]. Thus, transcriptional effects of DHA on SRXN expression in hippocampal cells are mechanistically very important since they ensure PRDX (and indirectly TXN) redox recycling even under prolonged prooxidant conditions [74,99,100].

DHA also modulates transcriptional activation of phase II detoxifying proteins such as heme oxygenase 1 (HO-1) and NAD(P)H quinone oxidoreductase 1 (NQO1) in nerve cells [62,64,101,102,103]. Previous studies have shown that upregulation of HO-1 or treatment with its downstream effectors and heme degradation products, biliverdin and CO, has protective effects in different rodent models of cerebral injury such as ischemia/reperfusion, stroke, and hemorrhage [103,104]. Heme oxygenase 1 (HO-1) serves as an inducible small enzyme that catalyzes the rate-limiting step in heme degradation, leading to the generation of biliverdin (rapidly converted to bilirubin by biliverdin reductase), carbon monoxide and iron ions. These products are potent direct antioxidants (biliverdin and bilirubin), but neuroprotection is mainly exerted by their functioning as anti-inflammatory, anti-apoptotic and pro-angiogenic factors [101,102,104,105]. On the other hand, the disturbances in proper HO-1 levels have been associated with the pathogenesis of some age-dependent nerve cell disorders, including neurodegeneration and macular degeneration [105,106]. 

NQO1 is a xenobiotic-metabolizing enzyme that catalyzes the oxidation of NAD(P)H into NAD(P) in the presence of quinones. Decreasing the NAD(P)H/NAD(P) ratio by increased transcription or enzyme activity upon binding of dunnione (an NQO1 substrate) promotes neuroprotection through modulation of NADPH oxidase (NOX)-derived ROS generation in mice [101,102,107]. Interestingly, both HO-1 and NQO1 detoxifying pathways share a common pattern of transcriptional regulation through activation of nuclear factor E2-related factor 2 (Nrf2) [62,101,102,103,105,107], as discussed in the next section. 

In summary, the effects of DHA on the different antioxidant systems described up to now strongly suggest a global strategy aimed at improving the ROS scavenging capacity of hippocampal cells by at least seven mechanisms: (1) by upregulating mitochondrial and cytoplasmic *Txn-Txnrd* gene expression and cellular TrxR activity; (2) by increasing transcriptional activation of mitochondrial peroxiredoxins; (3) by ensuring reactivation of hyperoxidated peroxiredoxins catalyzed by sulfiredoxin; (4) by upregulating genes involved in the biosynthesis of glutathione and reduction of GSSG; (5) by upregulating *Gpx4* gene expression and GPx4 enzyme activities to improve the cellular capability to recover oxidized phospholipids directly within cellular membranes; (6) by increasing levels of sentinel mRNA at dendritic locations to facilitate local response to oxidative threats and (7) by modulating transcriptional activation of inducible HO-1 and NQO1.

## 8. DHA: The Ultimate Indirect Antioxidant

Cellular protection against oxidative and electrophile toxicities is accomplished by two types of antioxidants: (i) direct antioxidants, which are redox active, short-lived, and are sacrificed in their antioxidant reactions and thereby need to be replenished or regenerated, and (ii) indirect antioxidants, which may or may not be redox active, but are transcriptional inducers of catalytically active proteins (mainly phase 2 detoxifying enzymes). Indirect antioxidants have long half-lives, are not consumed in the antioxidant reaction, and are often involved in regeneration of direct antioxidants. These two cytoprotective systems are related in complex functional crosstalk, i.e., (a) some direct antioxidants are required for the catalytic functions of redox proteins and (b) several antioxidant proteins participate in the synthesis and/or homeostasis of direct antioxidants. Direct antioxidants are diverse and common in nature but, importantly and less explored, many inducers of cytoprotective proteins have been isolated from edible plants, e.g., sulforaphane from broccoli and curcumin from turmeric [108,109]; ergothioneine, polyphenols and flavonoids from a variety of edible mushrooms [110,111] and DHA (but not arachidonic acid, the most abundant n-6 LCPUFA, or eicosapentaenoic acid, EPA, the second important n-3 LCPUFA) from different sources, mainly marine fish and invertebrates [112,113].

Thus, cumulative evidence points to DHA as the ultimate member of the list of natural indirect antioxidants. It is widely accepted that the nuclear factor erythroid-related factor 2 (Nrf2) is a master regulator of transcriptional activation of antioxidants in different tissues, including the brain [101,107,114]. Under physiological conditions, Nrf2 is bound to its repressor Kelch-like ECH-associated protein (Keap1), a ubiquitin ligase adaptor that functions as a cytoplasmic oxidative stress sensor. Upon activation, Nrf2 dissociates from Keap1, translocates to the nucleus and binds to the antioxidant response element (ARE) sequence located at the promoter region of some cytoprotective antioxidant genes essential for neuronal survival [101,102,104,107]. Most genes found to be transcriptionally regulated by DHA in nerve cells, including hippocampal, are amongst the list of genes containing ARE sequences and regulated by Nrf2, such as those encoding for GPx4, GR, GCLC, TXNRD, TXN, SRXNs, HO-1 and NQO1 [62,101,102,104,115,116]. Indeed, different studies have linked the antioxidant cytoprotective effect of DHA to the transcriptional regulation of gene expression through the Nrf2/Keap1/ARE pathway [102,103,115].

**Figure 5:**
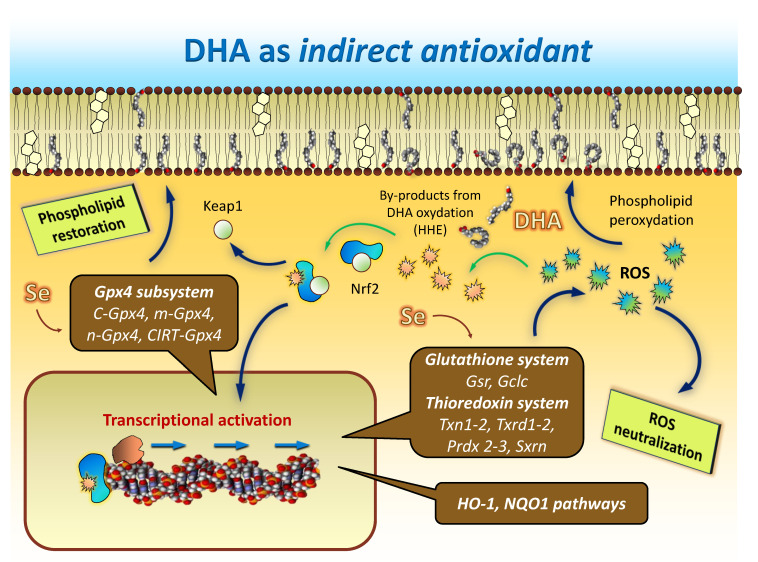
Hypothetical model illustrating how DHA accomplishes indirect antioxidant functions. Under challenging oxidative conditions, a fraction of DHA undergoes non-enzymatic oxidation, and DHA-derived reactive aldehydes such as 4-hydoxi-2-hexenal (HHE) are generated. This by-product activates Nrf2 transcription factor, which translocates to the nucleus and binds to ARE sequences in the promotor regions of different genes belonging to glutathione, thioredoxin, HO-1 and NQO1 antioxidant systems and pathways to trigger their transcriptional activation. By producing the set of mRNAs encoding for the different *Gpx4* isoforms, this regulated process not only improves the overall cellular antioxidant defense, but also the capability to recover oxidized phospholipids directly within nerve cell membranes.

DHA itself is not a ligand for Nrf2, so one important issue that remains to be determined the endogenous ligand underlying the transcriptional responses elicited by DHA. One recent observation indicates that low but significant levels of the specific DHA-derived peroxyl radical 4-hydroxy-2-hexenal (HHE) [66,117,118] are generated upon DHA supplementation [43] just before changes in gene expression are observed. Further, 4-hydroxy-2-hexenal has been demonstrated to be an activator ligand of Nrf2 [119], and recent studies in non-neuronal tissues have shown that, by generation of HHE, DHA stimulates transcription of antioxidant and phase II detoxifying enzymes through activation of Nrf2 [101,120]. These findings have been interpreted as HHE providing the signal to trigger the DHA-induced transcriptional regulation [44]. In line with these observations, we have proposed a plausible hypothesis for brain parenchyma that, under oxidative conditions, a fraction of DHA (either unesterified or phospholipid-bound) may undergo non-enzymatic oxidation to yield HHE, which, in turn, would activate a Nrf2-initiated transcriptional program (Figure 5). 

The result of such multigenic activation is aimed at providing oxidative resistance to DHA and other abundant LCPUFAs (e.g., arachidonic acid) in brain phospholipids Figure 5). The loss of efficiency of such homeostatic mechanisms likely underlies the general evidence for LCPUFA (particularly DHA) depletion during normal aging and, especially, in neurodegenerative diseases. 

## 9. Selenium Is an Absolute Requirement for Selenoprotein Biosynthesis

**Figure 6:**
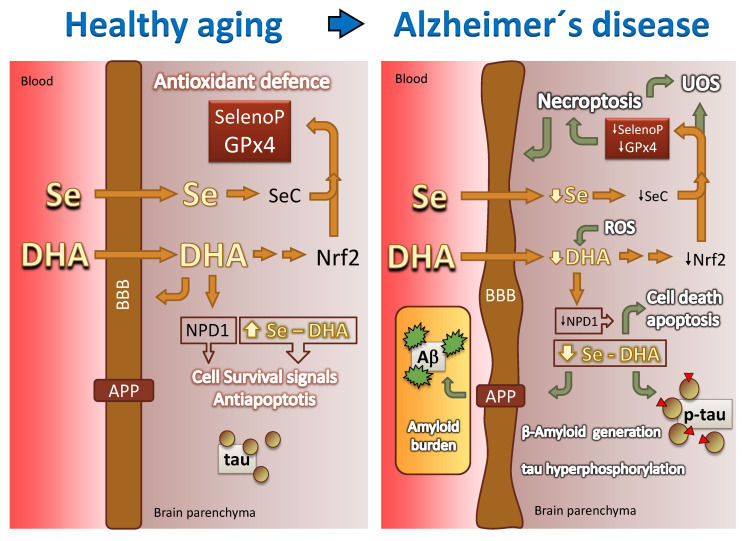
Graphical hypotheses on how changes in brain DHA and Se participate in healthy aging and in AD neurodegeneration. In AD a series of events reduces brain availability of DHA and Se. At least in part, this is due to impaired transport through the BBB and accumulation of reactive species. The reduced bioavailability of these factors triggers a cascade of cellular events, eventually leading to neuronal death, necroptosis, tau toxic amyloid β generation, amyloid burden and tau protein hyperphosphorylation. For details refer to Section 9, Section 10 and Section 11. BBB: blood–brain barrier; UOS: uncontrolled oxidative stress; APP: amyloid precursor protein. Green arrows denote deleterious events.

The ability of brain cells to maintain physiological levels of ROS well below UOS depends on their ability regulate the local biosynthesis of components of the antioxidant defense machinery (Figure 6). In the case of glutathione and thioredoxin systems, a limiting factor is the availability of selenocysteine (SeC), which, in turn, depends on selenium concentration. The concentration of selenium in the brain is very low (~0.03 μg g^−1^ wet tissue) [121], and the nerve cells have an absolute requirement for this micronutrient. Se depletion has been associated with different neuropathologies including the most prevalent Alzheimer’s disease (Figure 6) and Parkinson’s disease, but also Huntington’s disease and epilepsy [121,122,123]. All selenoproteins are characterized by the presence of one or two SeC residues in the active center to perform redox reactions [124]. SeC is an amino acid very similar to cysteine, but incorporates selenium instead of sulfur in the specific group. Selenoprotein synthesis is modulated by defined mechanisms that control gene transcription, RNA processing, translation and post-translational steps of protein biosynthesis [124]. Therefore, the physiological functions of selenoproteins strictly depend on the presence of Sec, and mutations of Sec to any other amino acid residue lead to enzyme inactivation [124]. In 1989, SeC was identified as the 21st amino acid, upon discovery of a novel tRNA (tRNASeC) coupled to SeC and recognition of UGA codons, which in normal proteins corresponds to a STOP codon, but in the formation of selenoprotein SeC is introduced without halting mRNA translation [125]. During translation of selenoproteins, the machinery is redirected to insert selenocysteine at UGA codons instead of terminating polypeptide synthesis. Some factors directly involved in this UGA translation process include specific secondary structure in the mRNA (SECIS), a unique tRNA (Sec-tRNA), a RNA binding protein (SBP2) and a specialized elongation factor (EFsec) [126]. Currently, 25 different SelenoP have been identified in the human proteome so far. The list includes the family of glutathione peroxidases already described, thioredoxin reductases (TXNRDS) and iodothyronine deiodinases (DIOs) [126]. Of note, in mammals there exists a hierarchy of Selenoprotein synthesis and expression in vivo when selenium availability is limited [127].

## 10. DHA and GPx4 in Aging and AD Brains

Aging is associated with a decline of physical and cognitive performances that leads to an increased risk of disease and death [128,129]. Aging occurs at different rates in different species and, within a particular species, there exist interindividual variations and even between different tissues from an individual [128,129]. Mechanisms of age-related changes are complex and involve different biological and environmental factors [130]. One widely accepted factor involved in aging-associated cognitive decline is the accumulation of reactive oxygen and nitrogen species (RONS) levels, which leads to damage of macromolecules (lipids, nucleic acids and proteins) ultimately causing cellular dishomeostasis [130,131,132,133]. The exact mechanisms of oxidative stress-induced aging are still not fully understood, but it is likely that toxicity of such reactive species occurs as a consequence of a decline of the brain’s ability to neutralize surplus RONS. Indeed, the increase in oxidative stress and inflammation by-products and metabolites has been reported during agin [134,135]. A relationship between increased oxidative stress biomarkers of lipids, proteins and DNA (i.e., malondialdehyde—MDA, 3-nitrotyrosine—3NT, 8-hydroxy-2′-deoxyguanosine—8-OHdG), and low cognitive performance has been observed in the elderly [136]. Besides MDA, other products of lipid peroxidation of LCPUFAs such as isoprostane F2 (F2-IsoPs) [137,138] and 4-hydroxinonenal [139] have been detected in the CSF during normal-to-pathological aging, reflecting the importance of lipid peroxidation in the course of aging. A number of studies have reported changes in lipid profiles of brain membranes during aging, which are consistent with underlying oxidative processes [134,135]. Current evidence has pointed to a membrane physicochemical modification as a result of aging, which is translated to membrane subdomains, such as lipid rafts [140,141,142,143]. In these structures, alterations in the levels of sterol esters, LCPUFAs (mainly DHA, AA), plasmalogens and sphingolipids have been reported as a result of aging [141]. Consistent with the pace of the aging process, the change in lipid composition of membrane lipid rafts is gradual [141,142]. Thus, in brain membranes, saturated fatty acids increase slowly at the age of 70 years, while there is a simultaneous decrease in the major n-6 and n-3 PUFAs (AA and DHA, respectively) peaking at the age of 80 years [134]. However, it has not been definitively elucidated whether these lipid changes belong to neurochemical traits of physiological aging, represent concurrent events at very early stages of emerging brain diseases or correspond to pathophysiological changes independent of age.

It is now well established that most, if not all, lipids classes are altered in AD brains [27,30,31,60,144,145,146]. The earliest studies in postmortem AD brains revealed lipid anomalies affecting both lipid classes and fatty acids in different brain areas including those associated (but not exclusively) with memory impairment such as the hippocampus and frontal cortex [30,31,146]. Subsequently, a wealth of studies has pointed to LCPUFAs and LCPUFA-containing phospholipids as particularly affected during AD, the depletion of DHA being the most common observation (Figure 6) [60,144,146]. The reduction of DHA is often accompanied by an increase in saturates and monounsaturates, which collectively causes the reduction of membrane unsaturation and peroxidability indexes. These changes may have a profound impact on the physiology and biophysics of nerve cell membranes [55,147,148,149]. Moreover, recent studies in human frontal and entorhinal cortices have demonstrated that such lipid changes are detectable in lipid raft microdomains, even at the earliest stages of AD, with an impact on their biophysical properties [29,53,54,150,151]. Interestingly, changes in the levels of saturated, monounsaturated and polyunsaturated fatty acids have also been recorded in the supernatant and brain-derived nanoparticle CSF fractions of AD [145]. DHA decreases in the supernatant fraction of AD CSF, while in the nanoparticle fraction, several polyunsaturated fatty acids derived from oxidation of n-3 and n-6 LCPUFAs (C20:2n-6, C20:3n-3, C22:4n-6, C22:5n-3) were increased. Similar changes were also observed for changes in the lipid composition of the two fractions during mild cognitive impairment [145] reinforcing the notion that changes in the CSF lipidome occur at early stages of AD. 

Overall, these data suggest that changes in lipid profiles and especially the decline in DHA levels affect brain tissue at different levels, from destabilization of membrane architecture and physiology to depression of endogenous antioxidant capacities, ultimately contributing to aging and to the development of Alzheimer’s disease (Figure 6).

A plausible link of lipid changes with UOS in AD is supported by numerous reports showing an oxidative signature in the cerebrospinal fluid of AD and also in MCI subjects [152]. In this regard, increased Iso-PG2, 4-HNE and MDA contents, have been consistently observed in the CSF of MCI and AD individuals [137,138,139,152]. Likewise, protein oxidative modifications are commonly detected in CSF of AD patients, such as S-cysteinylation, S-cysteinylglycinylation, S-glutathionylation as well as nucleic-acid-derived 8-hydroxy-2′-deoxyguanosine (8-OHdG) [45,68,153]. Protein nitration is another widely recognized marker of protein oxidation, and numerous studies support the idea that nitrosative stress contributes to neurodegeneration in AD [154,155]. An increase of glycation, oxidation and nitration adduct residues and free adducts has also been reported in the CSF of subjects with Alzheimer’s disease and MCI [154,155]. 

Most studies have demonstrated a depletion of n-3 series LCPUFAs, mainly docosahexaenoic acid (DHA) in postmortem AD brain [30,31,146]. 

As previously discussed, an essential master key in the preservation of brain lipidome is GPx4. GPx4 is expressed in glial cells and neurons throughout the brain, though its activity seems to exhibit regional differences. In healthy rat brain, the caudate-putamen and the substantia nigra show the highest activities, while the lowest activity was observed in corpus callosum [156]. Changes in the activity of peroxidases, including GPx4, have been observed in the human brain of both AD and PD patients, suggesting that decreased GPx4 activity is involved in these degenerative processes (Figure 6) [157,158,159]. GPx4 overexpression protects cortical neurons from oxidative injury and amyloid toxicity [160]. Genetic studies in animal models have been hampered because homozygous disruption of the GPx4 gene results in early embryonic lethality [78]. However, a direct relationship between GPx4 and AD has been obtained in heterozygous knockout mice (GPx4+/−), where increased γ-secretase activity and amyloid burden as well as formation of senile plaques were demonstrated [161]. 

The observation that hippocampal *Gpx4* gene expression is modulated by DHA in a genotype-related manner is also relevant. Our recent results in a transgenic APP/PS1 model of AD revealed that DHA upregulates all splicing variants of the gene, including the intron-retaining mRNA. However, the magnitude of this effect was isoform- and diet-dependent [44]. Thus, it was observed that the conditions imposed by a DHA-deficient diet were augmented to a greater degree than in the wild-type littermates. The effect was not observed for animals fed with DHA diets, which suggests that the combined stressing conditions of amyloid β accumulation (due to APP and PS1 transgenes overexpression) and the insufficient supply of DHA to the brain reinforce *Gpx4* expression (and especially the CIRT isoform) as an extreme adaptive strategy to preserve membrane DHA under strong adverse conditions [46]. 

Further, studies in GPx4BIKO mice (a mouse model with a conditional deletion in neurons of the forebrain of GPx4) have shown significant hippocampal neurodegeneration as well as deficits in spatial learning and memory function [162]. Similar results have been obtained using GPx4 inhibitors such as the Ras-selective lethal small molecule 3 (RSL3) [163]. Notably, maneuvers decreasing or abolishing adult brain GPx4 activity trigger a process known as ferroptosis, a type of programmed cell death dependent on iron and characterized by accumulation lipid peroxides secondary to decreased GPx4 activity (Figure 6) and often accompanied by ERK activation and neuroinflammation [67,164,165]. Gpx4-deleted mice do not survive past embryonic day 8, indicating that protection from ferroptosis is essential for normal mammalian development [81,166]. Ferroptosis is triggered by endogenous lipid peroxide designed apoptosis-inducing factor (AIF), resulting from 12/15-lipoxygenase-derived lipid peroxidation, which inhibits GPx4 function [167]. The precise identity of the 12/15-lipoxygenase-derived mediator of AIF activation is, as yet, unknown, but a possible candidate is 12(S)-hydroperoxyeicosatetraenoic (12S-HpETE). The exact pathway(s) underlying ferroptosis process have not been fully elucidated, but different reports have shown that AIF-mediated ferroptosis is initiated by a failure in glutathione antioxidant system defense, resulting in uncontrolled lipid peroxidation and ultimately cell death [164,165]. This occurs through two different (but not exclusive) mechanisms: (1) inhibition of *x*_c_^−^, the cysteine-glutamate antiporter, which limits the availability of cysteine and glutathione required for the function of GPx4, and (2) covalent binding of electrophilic lipoxygenase-mediated PUFA-derived inhibitors to the nucleophilic selenocysteine at the active site of GPx4 [164,165,168], as has been shown for the ferroptosis inducer (1S, 3R)-RSL3 [163,169,170].

As discussed above, transcriptional activity of the Nrf2/ARE pathway is at the basis of cellular antioxidant defense. This system has been shown to be effective at blocking neurotoxicity resulting from intracellular calcium overload, excitotoxicity, disruption of the mitochondrial electron transport chain, glutathione depletion and lipid peroxidation [104,107,114]. It is widely accepted that ARE-driven genes are preferentially, but not exclusively, activated in astrocytes and that Nrf2-ARE activation in glial cells not only precludes local oxidative damage but also confers protection to neighboring neurons [106,107,114]. Studies performed in different species have associated Nrf2 expression with longevity and maximum species lifespan [104]. A significant reduction of Nrf2 signal by aging has been demonstrated in different cell types, including nerve cells [104]. Age-related changes in the Nrf2 regulatory system involve increased expression of Keap1 and Bach1, which behave as endogenous repressors of Nrf2, leading to the inhibition of Nrf2 activity. Downstream effects of this inhibition in nerve cells include increased ROS generation and autophagy deregulation and reduction of injury-induced neurogenesis, proapoptotic protein, necroptosis and downregulation of antioxidant gene expression (Figure 6) [104]. This last effect has an enormous impact on the ability of DHA to induce restoration of oxidized phospholipids in nerve cell membranes thereby ensuring integrity and permeability.

The involvement of Nrf2 in the development of AD remains inconclusive, although most current evidence suggests an association with the disease progression. Thus, several studies have shown the increased expression of Nrf2 targets, including HO-1 and NAD(P)H: quinone oxidoreductase, during AD progression [106,107]. In glial cells, a potent upregulation of HO-1 has been shown in AD patients and subjects with MCI [105]. In addition, inhibition of HO-1 has been reported to improve behavioral anomalies in transgenic mouse models of AD [171,172]. Thus, it appears that increased activity of the Nrf2/HO-1 pathway is associated with AD. However, a detailed study in main affected brain regions of AD disclosed that nuclear Nrf2 expression is indeed decreased [173]. It is now accepted that Nrf2 is upregulated in the early stages of AD by β-amyloid-induced ROS, but starts to decrease as the disease progresses (Figure 6) [106].

## 11. Selenium and GPx4: The Fundamental Association

Selenium (Se) is a cofactor considered an absolute requirement for the synthesis of selenoproteins with essential roles in the brain tissue related to redox signaling, antioxidant defense systems and cell-mediated immune responses [115,174]. Although this metal is essential for brain homeostasis, it is scarce compared to other tissues [175]. Further, Se concentrations in the brain are heterogeneous both transversally, i.e., gray matter contains higher Se levels compared to white matter, and regionally, i.e., Se levels in rat brain were observed to be highest in the cerebellum and cerebral cortex and lowest in brainstem and spinal cord [176]. Surprisingly, brain Se distribution also appears to vary between species. Thus, in human brain, selenium concentration displays highest levels in the putamen, parietal inferior lobule and occipital cortex and lowest concentrations in the medulla and cerebellum [177], which notably differ from rat brain. Notably, results from the rat brain selenoproteome indicate that there exist priorities in brain selenium uptake and retention when Se supply is limited, and this is reflected in a well-established brain-specific hierarchy in response to Se deficiency [176]. Selenium and aging are closely linked. Thus, a recent proteomic study has demonstrated a strong interplay between selenium, selenoproteins and replicative senescence with a 72% overlap between the impact of senescence and selenium [178]. Further, several studies have suggested that the content of selenium in the elderly is significantly reduced, and this apparently accelerates the aging process [178]. Several studies have reported a positive association between selenium status and cognitive performance in older adults [179,180,181]. Moreover, in a recent randomized pilot study, Cardoso and collaborators [182] have reported that daily supplement with one Brazilian nut (corresponding to about 280 µg Se/day) over 6 months was associated with improved cognitive performance when given to patients with mild cognitive impairment. 

Studies in human brain have suggested that selenium status might be associated with increased oxidative-stress-mediated neurodegeneration and impaired cognitive function in AD [121,183]. During Alzheimer’s disease, decreased selenium levels have been observed at the temporal and hippocampal brain regions, while those in the amygdala were increased [183,184,185]. Alterations in cerebrospinal levels of selenium and its relationship with the expression and activity of GPx4 have been proposed as indicators of the processes underlying AD progression [183,186]. It has also been observed that selenium co-supplementation for 12 weeks improved cognitive function in AD patients [187]. In line with this, early Se treatment has also been shown to be neuroprotective after traumatic brain injury [188] which points to a modulatory Se-induced response against oxidative injuries. 

In general, Se supplementation studies in vitro are in agreement with the observations in brains from MCI and AD subjects. Thus, a number of reports have shown that selenium compounds prevent copper-and iron-mediated oxidative DNA damage due to metal binding, thus providing a possible additional antioxidant mechanism [189,190]. Selenite administration prevents secondary pathological effects including reduction of apoptotic cell death and neuronal destruction in the cortex and hippocampus of traumatic brain injury in models of cerebral ischemia [191]. The mechanisms of Se neuroprotection appear associated with modulation of Ca^2+^ influx via ion channels, anti-inflammatory effect by abrogation of microglia invasion and biosynthesis stimulation of antioxidant selenoproteins in the brain [192,193]. Additionally, it has been observed that selenoproteins prevent metal-mediated β-amyloid aggregation and inhibit the aggregation of tau protein induced by zinc [194,195]. It has shown that selenoprotein M induces a decrease in tau phosphorylation and reduces β/ϒ-secretase activities by modulating ERK signaling [196]. In addition, selenium supplementation prevents the Aβ peptide-induced neurotoxicity in rat primary hippocampal neurons [197]. In double knock-in transgenic mice (APP/PS1), administration of Se-enriched diets causes a decline in Aβ plaque deposition as well as a decrease in DNA and RNA oxidative damage, these effects being associated with increased GPx activity [185]. In Tg2576 mice (another transgenic model of familial AD), Se deficiency induces a two-fold increase in Aβ plaques compared to Se-adequate diets [172]. Selenite administration in a rat AD model also showed reduction of oxidative damage and morphological changes in the hippocampus and cerebral cortex, along with attenuation of cognitive deficits [198,199]. Further, a direct relationship between selenomethionine (SeMet), the major component of dietary Se, and GPx activity has been demonstrated for protection against Aβ-induced neuronal death (and also against other oxidative insults such as iron/H_2_O_2_-mediated toxicity) [200]. On the other hand, both in cell cultures and tau transgenic animal models, sodium selenate treatment reduced tau phosphorylation through specific activation of phosphatase PP2A [198,199]. In addition, selenomethionine treatment of AD transgenic mice results in a decrease in the concentration of total and phosphorylated tau and inflammatory markers and improved cognitive performance [201]. Therefore, it seems that poor selenium homeostasis and glutathione peroxidase activity are linked to oxidative brain damage and likely involved in the development of AD (Figure 6), while Se supplementation improves brain defense against oxidative injuries provoked by amyloid plaque burden and reduces tau hyperphosphorylation.

It is envisaged that Se levels in the cerebrospinal fluid (CSF) might provide insights into potential oxidative brain damage and neurodegenerative processes. However, results obtained on CSF selenium concentrations in humans are inconsistent. Thus, some studies have reported increased selenium levels in the of CSF patients with AD [202,203], while other publications fail to detect significant changes [188,204]. Selenium is transported to the brain by the action of selenoprotein P (SelP) at the BBB. Selenoprotein P (SEPP1) delivers selenium to the brain by binding to a surface receptor, apoER2, a member of the lipoprotein-receptor family [205]. Some studies have observed that deletion of selenoprotein P resulted in sharp decreases in brain (and testis) selenium concentrations [206,207]. A recent study assessing CSF selenium species, i.e., selenite (Se(IV)), selenate (Se(VI)), selenomethionine (Se-Met) and selenocysteine (SeC) indicate that rather than total Se, alterations in some inorganic forms such as selenite can satisfactorily predict conversion to AD in persons with MCI [208]. The increase in selenium species and seleno P in the CSF of AD patients strongly indicates that destabilization of this barrier is part of the neurodegenerative process (Figure 6), as others have proposed [209,210,211]. Obviously, further in-depth studies on the mechanisms of Se transport across the BBB and its metabolism in the brain are necessary to substantiate the relationship between Se levels in MCI and progression to AD and probably other oxidative stress-related neurodegenerative disorders. In line with this, it is important to highlight that DHA contributes to preserving the integrity of the BBB (Figure 6). At least in some models, DHA has been proven to attenuate disruption of the BBB by experimentally induced focal ischemic stroke [208]). It remains to be established whether DHA might also promote BBB preservation in slowly developing neurodegenerative processes. 

## 12. Conclusions

DHA is a pleiotropic molecule that modulates the physicochemical properties and architecture of neuronal plasma membrane, but beyond structural functions, DHA is also involved in multiple facets of neuronal biology, from regulation of synaptic function to neuroprotection and modulation of gene expression. In the pro-oxidant environment of brain parenchyma, DHA is highly susceptible to oxidation, but the brain neurochemistry has evolved to provide exceptional protection against LCPUFA peroxidation. The recent findings have disclosed the ability of DHA to behave as an indirect antioxidant. As such, DHA regulates a full transcriptional program that potentiates the antioxidant defenses of nerve cells, including the glutathione/glutaredoxin and thioredoxin/peroxiredoxin systems as well as Heme oxygenase 1 and NAD(P)H:quinone oxidoreductase pathways. Of these, the most elaborated is perhaps the upregulation of *Gpx4,* the gene encoding for the phospholipid-hydroperoxide glutathione peroxidase, also named glutathione peroxidase 4, the main enzyme capable of reducing oxidized phospholipids in-membrane. Noticeably, DHA also upregulates a cytoplasmic intron-sequence-retaining transcript from the *Gpx4* gene, which behaves as a “sentinel RNA”. This expands the antioxidant protection of neuronal membrane by GPx4 away from conventional splicing in the nucleus, as in the case of dendrites. Efficient and rapid synthesis of GPx4 isozymes in the brain strongly depends on the in situ availability of selenium, the transport of which into the brain is the rate-limiting factor for selenocysteine insertion into the catalytic domain of GPx4. 

Overall, the present review adds to the complexity and diversity of DHA actions in the brain, these ranging from precise determinants of nerve cell biophysical properties to transcriptional regulation of endogenous antioxidant capacity. The finely tuned homeostasis of DHA in nerve cells is paramount in the context of normal and pathological aging, particularly in Alzheimer’s disease. 

## Figures and Tables

**Table 1:** Simplified compilation of DHA actions in nerve cells. PE: phosphatidylethanolamine. LTP: long-term potentiation.

Function	Examples	References
Bilayer Asymmetry	DHA-enriched PE: Inner leaflet >> Outer leaflet	[3,5,6,8,9,15]
Organization of Membrane Domains	Segregation of lipid rafts and non-raft microdomains	[5,6,9,10,15]
Biophysical Properties	Membrane fluidity, thermodynamic of lipid–protein interactions	[5,6,10,53,54,55]
Nerve Cell Signaling	Neuroprotectin D1/PI3K/Akt pathway	[4,39,41,42]
Neurodevelopment	Fetal and Postnatal Brain accretion	[3,21,56]
Neuromodulation	Synaptogenesis, LTP, dendritogenesis, neurotransmission	[3,4,18,19,20,21,22,23,24,25]
Anti-inflammatory Mediators	Docosanoids, elovanoids	[4,39,40,47]
Modulation of Apoptosis	Induction of anti-apoptotic Bcl-2 family of proteins	[4,40,41,47,48,49,51,52]
Regulation of Lipid Metabolism	Cholesterol biosynthesis, phospholipid remodeling	[57,58,59]
Neuroprotection	Processing of amyloid precursor protein (APP)	[7,26,35,36,37,50,54,60]
Modulation of Gene Transcription	Interaction with PPAR/RXR/Nrf2 transcription factors	[22,61,62,63,64]

## Abbreviations

**12S-HpETE**	12(S)-hydroperoxyeicosatetraenoic
**3NT**	3-nitrotyrosine
**8-OHdG**	8-hydroxy-2′-deoxyguanosine
**AA**	Arachidonic acid
**AIF**	Apoptosis-inducing factor
**APP**	Amyloid precursor protein
**ARE**	Antioxidant response elements
**BBB**	Blood–brain barrier
**BDNF**	Brain-derived neurotrophic factor
**c-GPx4**	Cytosolic glutathione peroxidase 4
**CIRTs**	Cytoplasmic intron-sequence-retaining transcripts
**CSF**	Cerebrospinal fluid
**DHA**	Docosahexaenoic acid
**EFsec**	Elongation factor
**EPA**	Eicosapentaenoic acid
**F2-IsoPs**	Isoprostane F2
**GCLC**	Glutamate-cysteine ligase
**GLRX**	Glutaredoxin
**GPx**	Glutathione peroxidase
**GSR**	Glutathione-S-reductase
**GST**	Glutathione-S-transferase
**HHE**	4-hydroxy-2-trans-hexenal
**HNE**	4-hydroxy-2-trans-nonenal
**HO-1**	Heme oxygenase 1
**Keap1**	Kelch-like ECH-associated protein
**L^•^**	Lipid radicals
**LCPUFAs**	Long-chain polyunsaturated fatty acids
**LOO^•^**	Lipoperoxyl radicals
**LOOH**	Lipoperoxide
**MCI**	Mild cognitive impairment
**MDA**	Malondialdehyde
**m-GPx4**	Mithocondrial glutathione peroxidase 4
**n-GPx4**	Nuclear glutathione peroxidase 4
**NPD1**	Neuroprotectin D1
**NQO1**	NAD(P)H quinone oxidoreductase 1
**Nrf2**	Nuclear factor erythroid-related factor 2
**O_2_^−^**	Superoxide anion
**OH^•^**	Hydroxyl radical
**ONOO^−^**	Peroxynitrite
**PE**	Phosphatidylethanolamine
**Prdx2**	2-Cys type peroxiredoxin gene
**PRDX2**	Cytosolic peroxiredoxin
**PRDX3**	Mitochondrial peroxiredoxin
**PS1**	Presenilin 1
**PUFAs**	Polyunsaturated fatty acids
**RCS**	Reactive carbonyl species
**RNP**	Ribonucleoprotein
**RNS**	Reactive nitrogen species
**ROS**	Reactive oxygen species
**RSL3**	Ras-selective lethal small molecule 3
**SBP2**	RNA binding protein 2
**SeC**	Selenocysteine
**SelenoP**	Selenoproteins
**SeMet**	Selenomethionine
**SRXN**	Sulfiredoxin
**TXN**	Thioredoxin
**TXNIP**	Thioredoxin interacting protein
**TXNRD**	Thioredoxin reductase
**UOS**	Uncontrolled oxidative stress
